# YwqL (EndoV), ExoA and PolA act in a novel alternative excision pathway to repair deaminated DNA bases in *Bacillus subtilis*

**DOI:** 10.1371/journal.pone.0211653

**Published:** 2019-02-06

**Authors:** Adriana G. Patlán, Víctor M. Ayala-García, Luz I. Valenzuela-García, Jimena Meneses-Plascencia, Pedro L. Vargas-Arias, Marcelo Barraza-Salas, Peter Setlow, Luis G. Brieba, Mario Pedraza-Reyes

**Affiliations:** 1 Departamento de Biología, Universidad de Guanajuato, Noria Alta, Guanajuato, Guanajuato, México; 2 Facultad de Ciencias Químicas, Universidad Juárez del Estado de Durango, Durango, Durango, México; 3 Department of Molecular Biology and Biophysics, UConn Health, Farmington, Connecticut, United States of America; 4 Langebio-Cinvestav Sede Irapuato, Km. 9.6 Libramiento Norte. Carretera Irapuato-León, Irapuato, Guanajuato, México; University of Massachusetts Medical School, UNITED STATES

## Abstract

DNA deamination generates base transitions and apurinic/apyrimidinic (AP)-sites which are potentially genotoxic and cytotoxic. In *Bacillus subtilis* uracil can be removed from DNA by the uracil DNA-glycosylase through the base excision repair pathway. Genetic evidence suggests that *B*. *subtilis* YwqL, a homolog of Endonuclease-V (EndoV), acts on a wider spectrum of deaminated bases but the factors that complete this pathway have remained elusive. Here, we report that a purified His_6_-YwqL (hereafter *Bs*EndoV) protein had *in vitro* endonuclease activity against double-stranded DNAs containing a single uracil (U), hypoxanthine (Hx), xanthine (X) or an AP site. Interestingly, while *Bs*EndoV catalyzed a single strand break at the second phosphodiester bond towards the 3'-end of the U and AP lesions, there was an additional cleavage of the phosphodiester bond preceding the Hx and X lesions. Remarkably, the repair event initiated by *Bs*EndoV on Hx and X, was completed by a recombinant *B*. *subtilis* His_6_-DNA polymerase A (*Bs*PolA), but not on *Bs*EndoV-processed U and AP lesions. For the latter lesions a second excision event performed by a recombinant *B*. *subtilis* His_6_-ExoA (*Bs*ExoA) was necessary before completion of their repair by *Bs*PolA. These results suggest the existence of a novel alternative excision repair pathway in *B*. *subtilis* that counteracts the genotoxic effects of base deamination. The presence of this novel pathway *in vivo* in *B*. *subtilis* was also supported by analysis of effects of single or multiple deletions of *exoA*, *endoV* and *polA* on spontaneous mutations in growing cells, and the sensitivity of growing wild-type and mutant cells to a DNA deaminating agent.

## Introduction

Deamination of DNA bases is one of the most common types of genetic insults in all organisms. Exocyclic amino groups in cytosine, adenine and guanine are particularly vulnerable to spontaneous or chemically induced hydrolytic events [1–5]. Deamination of cytosine, adenine and guanine generates uracil (U), hypoxanthine (Hx) and xanthine (X), respectively, and if not removed from DNA, these lesions promote transition mutations including, CG to TA, AT to GC and GC to AT, respectively [6]. To counteract the adverse effects of U, bacteria and mammals rely on repair proteins termed uracil DNA glycosylases (Ung) [7], which catalyze the cleavage of the glycosidic bond that connects U with the deoxyribose moiety, generating an apurinic/apyrimidinic (AP) site; this non-coding lesion is further processed by components of the canonical base excision repair pathway (BER) [7,8,9].

Some bacteria and archaea also have a repair protein able to recognize and hydrolyze double-stranded DNA containing a wide spectrum of genetic lesions, including uracil, additional deaminated bases, AP sites, mismatches, flap structures and pseudo-Y structures [10–13]. In *E*. *coli*, this enzyme, encoded by the *nfi* gene (EC: 3.1.21.7), has been termed endonuclease V (EndoV) [14,15]. This Mg^2+^-dependent enzyme catalyzes the incision of the second phosphodiester bond towards the 3' end of the lesion [13], thus constituting the first step of an Alternative Excision Repair (AER) pathway [6,16]. Fundamental aspects of catalysis, function and structure of EndoV homologs from distinct organisms have been compiled in an excellent recent review [17].

The genome of the Gram-positive bacterium *Bacillus subtilis* possess a protein termed YwqL whose amino acid sequence shares 51% identity with that of EndoV (Nfi) from *E*. *coli* [18]. It has been shown that YwqL (hereafter *Bs*EndoV) plays a more prominent role than Ung in protecting *B*. *subtilis* from the cytotoxic and genotoxic effects of spontaneous and induced factors that promote DNA deamination in this bacterium [18]. Additionally, starved, non-growing *B*. *subtilis* cells lacking *ung* and *ywqL* (hereafter *endoV*) increased their mutation frequency in response to accumulation of DNA lesions [19)]. A recent report revealed that in addition to Ung and *Bs*EndoV, *B*. *subtilis* employs Aag, an alkyl-adenine DNA-glycosylase, to help counteract the noxious effects of base deamination [20].

The downstream steps completing the hydrolytic event catalyzed by EndoV on different DNA substrates are currently a matter of investigation. However, the proofreading and polymerase activities of DNA polymerase I (Pol I) are involved in this pathway in *E*. *coli* [6, 21, 22]. This proposed mechanism postulates that Pol I recognizes the nick generated by EndoV and uses its 3'➔5' exonuclease activity to remove a stretch of DNA containing the lesion to generate a new 3' -OH terminus. The DNA gap is subsequently filled by Pol I and the repair process is completed by a DNA ligase [6,21,22]. However, genetic and structural evidence have revealed the lack of proofreading (3’➔5’ exonuclease) activity in DNA Pol Is from members of the genus *Bacillus* [23,24], thus, ruling out the existence of this simple repair pathway in *B*. *subtilis*.

In *B*. *subtilis*, PolA (*Bsu*PolA), the homolog of Pol I of *E*. *coli*, works in concert with the BER and nucleotide excision repair (NER) systems to maintain the genome integrity of actively replicating cells [6,25,26]. Further evidence has implicated this enzyme in translesion synthesis (TLS), interacting with low fidelity DNA-polymerases of the Y-family [27], as well as in stimulating formation of mutations that allow nutritionally stressed cells to escape from growth-arresting conditions [28].

In *Salmonella enterica* Pol I is essential for growth [29], and in Pol I-deficient *E*. *coli*, this growth defect is only manifested in rich medium [30]. In contrast, PolA is not essential for efficient *B*. *subtilis* growth, a difference presumably attributed to the 5'➔3' exonuclease activity present in a paralogous *B*. *subtilis* protein termed YpcP. In support of this notion, a double mutant *polA ypcP* is not viable [27,31,32] and recently the participation of YpcP in an alternative route for the repair of UV-photoproducts initiated by the UV-endonuclease YwjD along with Y-family DNA polymerases was suggested [33]. Although the function of *Bsu*PolA has been extensively determined at the genetic and physiological levels, to our knowledge, evidence reporting the contribution of this enzyme to *in vitro* DNA repair processes have remained elusive.

In this work, we report the cloning, heterologous synthesis and purification of a His_6_-YwqL protein from *B*. *subtilis* (*Bs*EndoV) that exhibited *in vitro* activity on synthetic ds-DNA substrates carrying U, Hx, X or AP sites. *Bs*EndoV catalyzed a single hydrolytic event downstream of the DNA strand containing U or an AP site generating a single DNA nick. In contrast, in DNA substrates containing Hx or X, the purified enzyme performed a double cleavage flanking both lesions. Remarkably, the intermediate products derived from *Bs*EndoV action on Hx or X substrates, but not those from action on U and AP substrates, were efficiently repaired by a pure recombinant *B*. *subtilis* His_6_-PolA (*Bs*PolA). However, the repair products resulting from hydrolysis on U or AP-containing ds-DNA by *Bs*EndoV required the 3'➔5' exonuclease activity of ExoA to excise these lesions prior to *Bs*PolA-dependent DNA synthesis. We also analyzed the effects of *endoV*, *exoA* and *polA* mutations, alone or in combinations, on spontaneous mutagenesis of growing *B*. *subtilis* cells, as well as cell killing by a DNA deaminating agent. Overall, our results suggest the presence of of a novel alternative repair pathway in *B*. *subtilis* that operates on deaminated bases and AP-sites employing the proteins YwqL, ExoA and PolA.

## Results

### Sequence and structure analyses of different EndoV enzymes and purification of *Bs*EndoV

**Fig 1:**
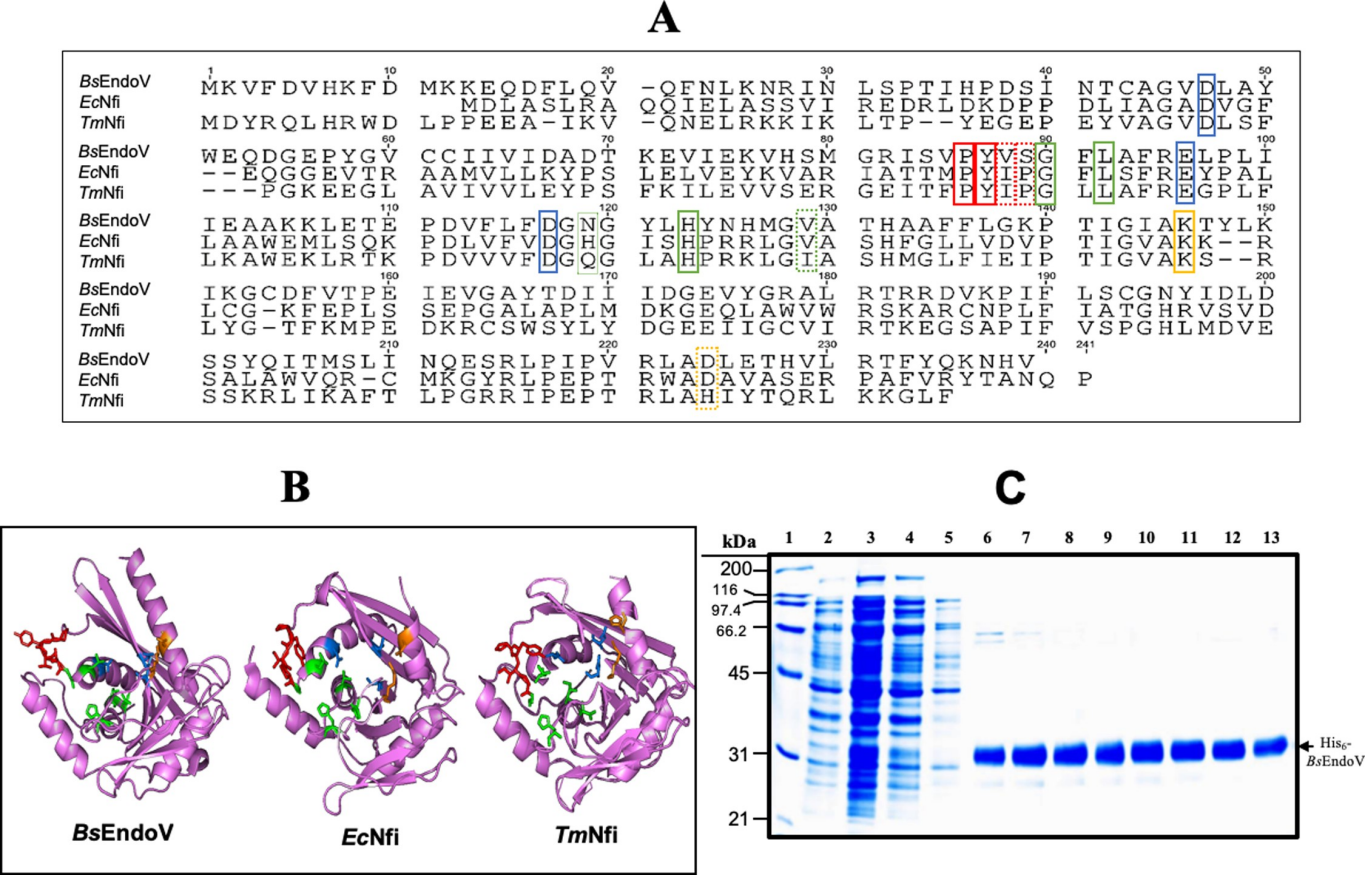
Comparison of different Endonuclease Vs and purification of *Bs*EndoV. (A) Amino acid sequence alignment of *Bs*EndoV with homologs from *E*. *coli* (Nfi) and *T*. *maritima* (Nfi). Identical residues involved in activity between enzymes are enclosed with solid lines; dissimilar residues are enclosed with dashed lines. Colors of amino acids denote: red—residues comprising the insertional wedge to stabilize flipping-out of Hx; green—residues involved in the damaged-base recognition pocket; blue—residues that bind Mg^2+^ necessary for endonucleolytic action; and orange—residues involved in forming the post-incision complex. (B) Tertiary structures (magenta) of EndoV homologs from *B*. *subtilis*, *E*. *coli* and *T*. *maritima*; PDB accession numbers 3HD0, 3GA2 and 4XPU, respectively. Amino acids involved in recognition, catalysis, and complex formation are depicted as sticks with the same color code as in (A). (C) SDS-PAGE analysis of *Bs*EndoV induction and purification by IMAC, all as described in Methods. Lane 1, molecular weight markers; lane 2, cell lysate of non-induced *E*.*coli* PERM1071 strain; lane 3, IPTG-induced extract of *E*. *coli* PERM1071; lane 4, flowthrough; lane 5, wash fraction and lanes 6 to 13, protein eluted from the Ni-NTA column with 250 mM imidazole. The purified protein from fraction 13 was employed for the DNA repair assays.

*B*. *subtilis* strains deficient for Ung and/or *Bs*EndoV exhibit, significantly decreased ability to contend with the genotoxic effects of spontaneous and induced DNA deamination, suggesting that these enzymes are important in maintaining genomic integrity in this bacterium [18]. The biochemical properties and substrate specificity of a purified recombinant Ung protein from *B*. *subtilis* were recently reported [34]. However, the demonstration that the protein product of the *endoV* gene possesses the capability to remove deaminated bases and other lesions from DNA has remained elusive. The mechanistic aspects governing the structure/function relationship of EndoV proteins have been explored in bacteria, although these studies have been focused mainly on the processing of hypoxanthine [35,36,37]. Of note, whereas the primary structure of *Bs*EndoV has 51 and 57% amino acid similarity with EndoV proteins from *E*. *coli* and *T*. *maritima*, respectively, significant differences in key amino acids involved in recognition and processing of the DNA lesion can be identified in *Bs*EndoV (Fig 1A). These modifications predicted significant structural changes in the wedge pocket recognition and DNA-protein stabilization domains of *Bs*EndoV (Fig 1A and 1B) that may impact its substrate recognition and catalytic properties. To investigate these possibilities, the *endoV* gene was amplified by PCR and the gene product expressed from the IPTG inducible T_5_ promoter of the plasmid pQE30 to generate a protein tagged with 6 histidines on its N-terminus. The optimum conditions for *endoV* induction, 0.25 mM IPTG for 3 h at 28°C, produced the maximum amount of the recombinant protein in soluble form. SDS-PAGE analysis showed the presence of a highly abundant soluble 31 kDa protein in cell extracts from cultures of *E*. *coli* PERM1071 induced with IPTG, but not from those that were not supplemented with this inducer (Fig 1C, lanes 2, 3). The induced cell extract was loaded onto a Ni-NTA-agarose column, and a protein with a molecular mass of ~31 kDa and high purity was eluted from the column with 250 mM imidazole as revealed by SDS-PAGE (Fig 1C, lanes 6–13).

### Endonuclease activity of His_6_-*Bs*EndoV on ds-DNAs containing U, X, Hx or an AP site

**Fig 2:**
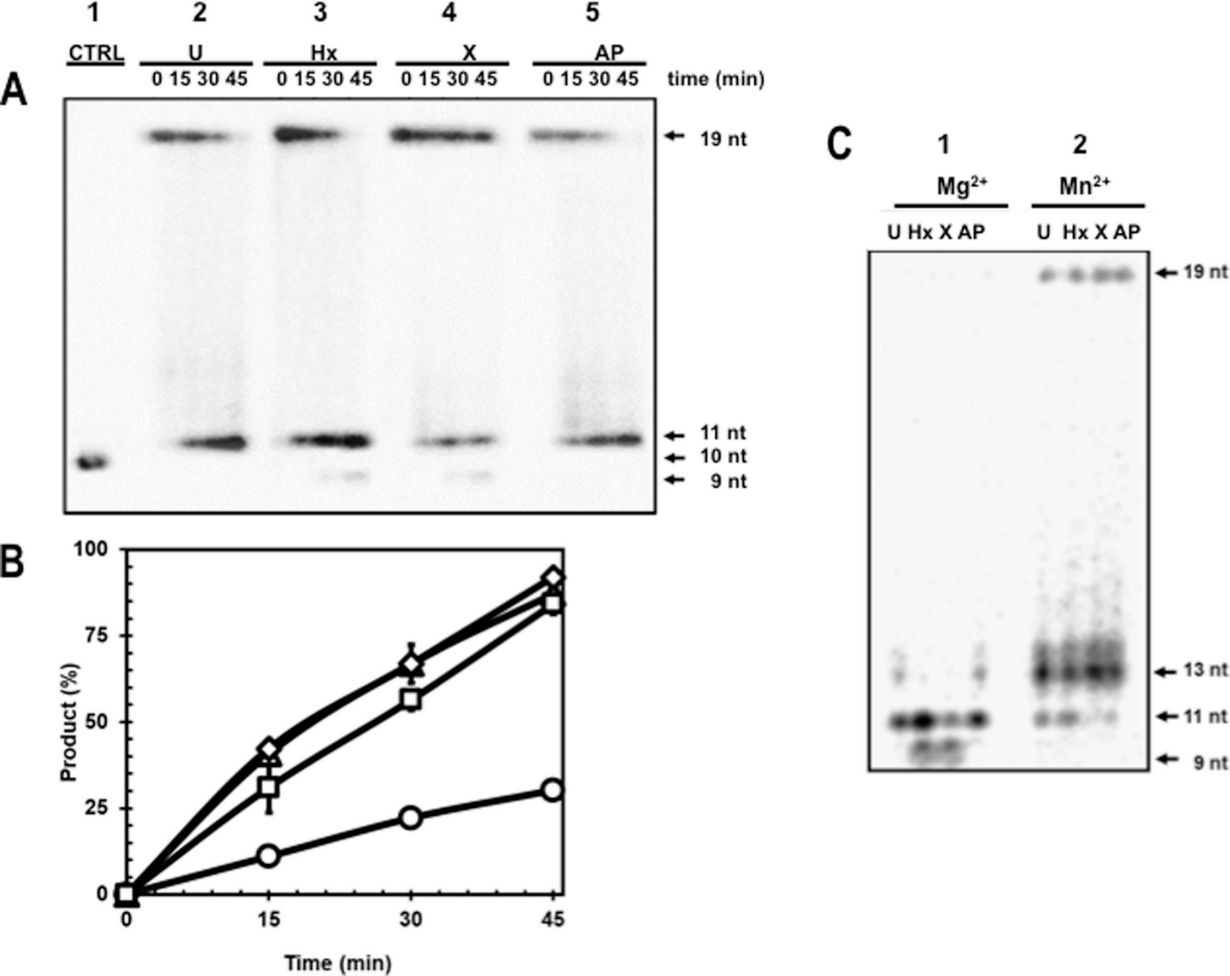
Differential processing of DNA lesions by *Bs*EndoV. **(A)** Ability of *Bs*EndoV to process different deaminated bases and AP-sites in ds-DNA. A [^32^P]-labeled 19-mer-DNA (at 10 nM concentration) containing a U (column 2), a Hx (column 3), an X (column 4), or an AP-site (column 5) were annealed to their complementary non-radioactive oligonucleotide and incubated separately with 100 nM of *Bs*EndoV in presence of MgCl_2_ as described in Methods, and samples were collected at indicated times. A control reaction (column 1) contained the labeled oligonucleotide with U incubated with commercial uracil-DNA glycosylase (Ung) and AP-endonuclease (Nfo) in order to estimate the size of the nicked products generated by *Bs*EndoV. All reactions were separated by denaturing electrophoresis, exposed and analyzed as described in Methods. **(B)** Densitometry analyses of the conversion of each substrate into product by *Bs*EndoV using radiolabeled ds-DNAs containing U (Δ), Hx (◊), X (○) or an AP-site (□). **(C)** Effects of the divalent ions Mg^2+^ (column 1) and Mn^2+^ (column 2) on the endonuclease activity of *Bs*EndoV on ds-DNAs containing deaminated bases or an AP site. U, uracil; H, hypoxanthine; X, xanthine and AP, apurinic/apirimidinic site.

EndoVs are promiscuous enzymes that can act on different DNA lesions depending on their origin [17]. Therefore, the enzymatic activity of *Bs*EndoV was tested against 5'-[^32^P] end-labelled ds-19-mer DNAs containing, uracil, hypoxanthine, xanthine or an AP-site. In these experiments, the reactions were supplemented with Mg^2+^ to optimize the endonuclease activity of *Bs*EndoV [17)]. To unambiguously establish the position of the phosphodiester bond hydrolysed by *Bs*EndoV, the 19-bp DNA duplex containing uracil was sequentially treated with commercial Ung to generate an AP site and subsequently with the AP-endonuclease Nfo, generating the expected 10-nt long radioactive ss-oligonucleotide (Fig 2A, column 1). As shown in Fig 2A (columns 2–5), *Bs*EndoV was also able to incise the four damaged substrates in a time-dependent fashion, mainly generating an 11-nt radioactive ss-oligonucleotide, strongly suggesting that the enzyme attacked the second phosphodiester bond downstream of these lesions. Of note, the 5'-end-radiolabelled ds-19-mer DNA free of lesions remained intact after 45 min of incubation with *Bs*EndoV (S1 Fig), thus confirming the specificity of this enzyme to operate over ds-DNAs carrying deaminated bases or an AP site. Remarkably, whereas *Bs*EndoV exclusively produced the 11-nt radioactive product with the substrates containing U or AP lesions (Fig 2; lanes 2 and 5), small amounts of an additional 9-nt long radioactive product (of the 11nt product) resulted from the processing of the 19-mer substrates carrying the HX and X lesions (Fig 2A; lanes 3 and 4). This DNA product, which could result from a double incision event or from a 3'➔5' exonucleolytic activity displayed by *Bs*EndoV, may potentially eliminate the stretch of DNA bearing Hx and X. Quantitation of the cleavage products by densitometry revealed that *Bs*EndoV processed uracil, hypoxanthine and AP lesions with approximately the same efficiency, but xanthine was nicked to a much lesser extent (Fig 2B and Table 1). These results revealed that *Bs*EndoV retains the promiscuous character of EndoV homologs and also suggests that downstream repair events may be different for HX and X than for U and AP sites.

**Table 1:** Percentage of cleavage of different lesions by *Bs*EndoV.

Lesion	^a^% of cleavage
**Uracil**	**87.1 ± 1.5**
**Hypoxanthine**	**92 ± 3**
**Xanthine**	**30.1 ± 1.6**
**AP-site**	**84.2 ± 2**

^a^The percent of remaining radioactive substrates following 45 min incubation with *Bs*EndoV were quantitated by densitometry in reference to non-enzymatically treated controls as shown in Fig 2B. Results are the average of three independent experiments ± SD.

EndoVs employ Mg^2+^ for efficient catalysis; as previously reported, this ion is responsible for activating a water molecule involved in hydrolysing the phosphodiester bond downstream of the recognized lesion [35,38]. Although Mg^2+^ is the natural cofactor for EndoV [17], this metal can apparently be substituted for Mn^2+^ [38]. Consequently, the effect of both metals on the substrate specificity of *Bs*EndoV were tested. Independent reactions containing 100 nM of purified *Bs*EndoV and the 19-bp DNA duplex substrates containing U, Hx, X or an AP site were incubated for 1 h at 37°C in reactions with MgCl_2_ or MnCl_2_. In reactions with Mg^2+^, *Bs*EndoV generated the expected pattern of repair products with the four damaged DNA substrates (Fig 2C column 1). These results corroborated the specific attack of the second phosphodiester bond located downstream of the four lesions tested as well as an additional incision repair event on the phosphodiester bond preceding the Hx and X lesions (Fig 2C, column 1). However, with Mn^2+^ present, the specificity of *Bs*EndoV on all 4 DNA substrates tested changed, as products larger than 11-nt, primarily 13 nt, were generated from all substrates (Fig 2C, column 2).

### Purification and DNA-Polymerase activity of a recombinant *B*. *subtilis* His_6_-PolA protein (*Bs*PolA)

**Fig 3:**
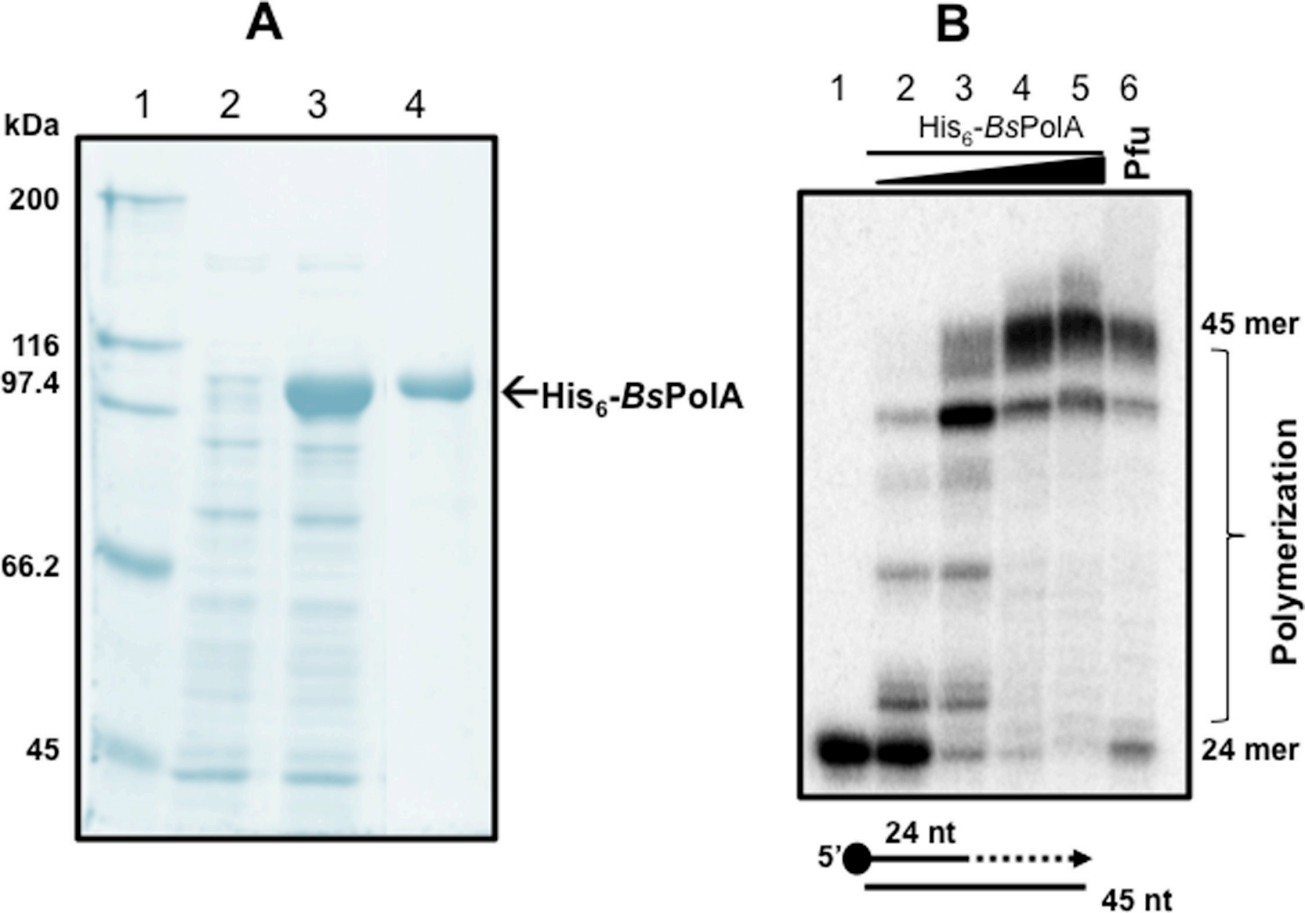
Purification and DNA polymerase activity of a recombinant *Bs*PolA. **(A)** SDS-PAGE analysis of His_6_-*Bs*PolA induction and purification by IMAC. *E*. *coli* PERM1367 was grown, induced and enzyme extracted and purified as described in Methods. Lane 1, molecular weight markers; lane 2, cell lysate of non-induced *E*. *coli* PERM1367 strain; lane 3, IPTG-induced extract of *E*. *coli* PERM1367; and lane 4, protein eluted from the Ni-NTA column with 300 mM imidazole. **(B)** DNA polymerase activity of *Bs*PolA *in vitro*. Primer extension reactions were conducted with 0, 1, 10, 100 or 1000 nM of *Bs*PolA (lanes 1–5) incubated at 37°C with a radiolabeled 24-mer-oligonucleotide hybridized with a non-radioactive 45-mer-oligonucleotide probe and supplemented with dNTPs as described in Methods. A positive control for DNA synthesis was carried out with *Pfu* DNA polymerase (lane 6). All reactions were separated by denaturing electrophoresis, exposed and analyzed as described in Methods.

In different microorganisms PolA participates in the post-incision/excision events of the canonical mismatch repair (MMR), NER and BER pathways, thus playing a central role in maintaining the bacterial genome free of damage [39]. To test the contribution of PolA to the post-incision repair events initiated by *Bs*EndoV on deaminated DNA *in vitro*, it was necessary to produce and purify a recombinant form of *Bs*PolA. This task was accomplished in an *E*. *coli* strain carrying a construct overexpressing a *his*_*6*_-*polA B*. *subtilis* gene from an IPTG-inducible promoter. In the presence of the inducer, this heterologous host generated high amounts of a soluble protein with the expected molecular mass of *Bs*PolA, ~100 kDa (Fig 3A, lane 3). The *Bs*PolA protein purified to homogeneity by metal-affinity chromatography (Fig 3A, lane 4) was functional, as evidenced by its capacity to catalyse the full extension of a 5'-[^32^P] labelled 24-nt primer hybridized to a 45-nt complementary strand (Fig 3B, Lanes 2–5). A positive control with commercial *Pfu* DNA polymerase, which also extended the radioactive primer, attested to the functionality of the polymerase assay employed in these experiments (Fig 3B, lane 6).

### *Bs*EndoV and *Bs*PolA eliminate Hx and X from DNA but not U and AP lesions

**Fig 4:**
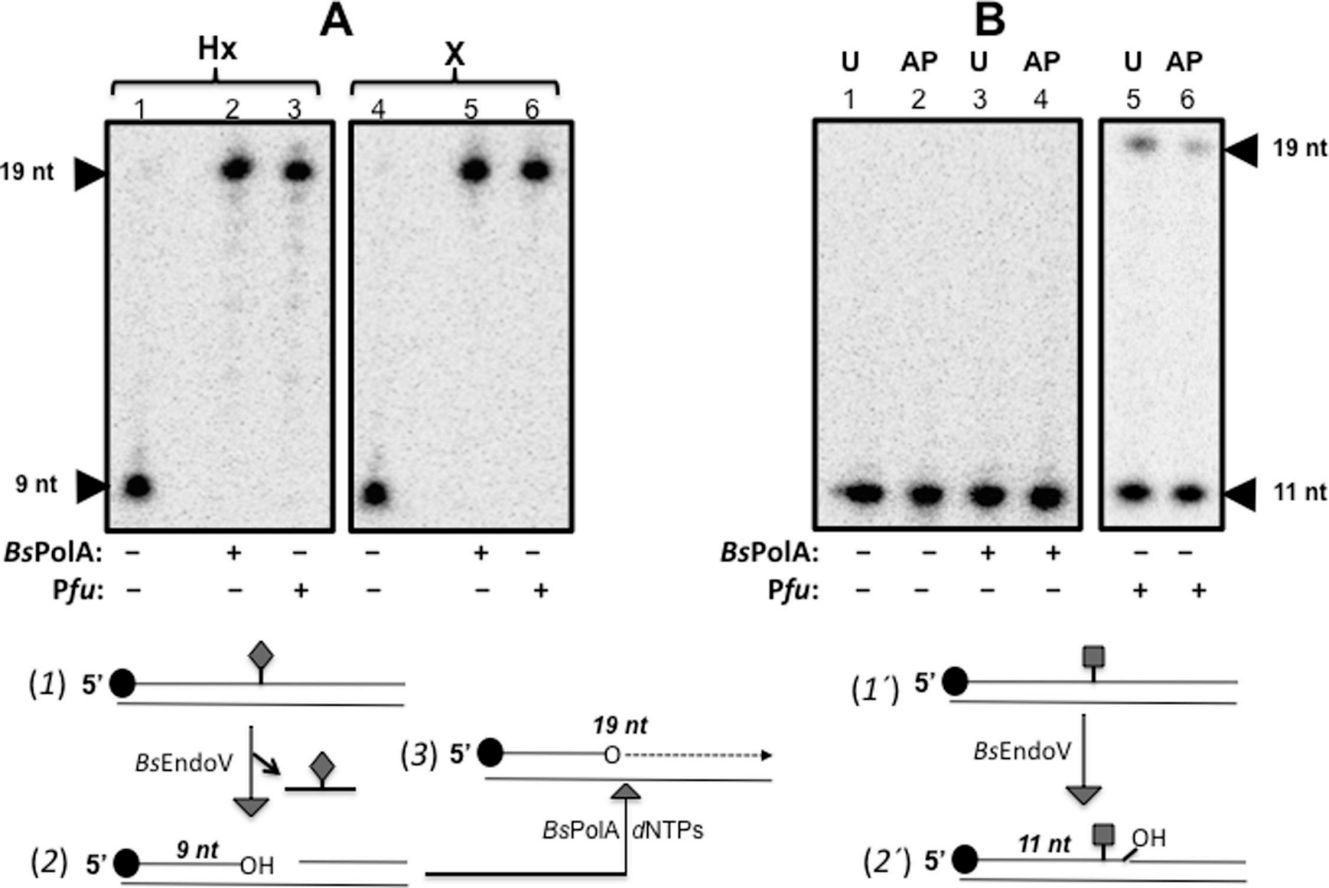
Ability of *Bs*PolA to recognize and polymerize repair-intermediates generated by *Bs*EndoV. **(A)** *Bs*EndoV-incised ds-DNA substrates carrying Hx (lanes 1–3) or X (lanes 4–6) were purified and employed as substrates for *Bs*PolA-dependent DNA synthesis. **(B)** *Bs*EndoV-incised ds-DNA substrates carrying U (lanes 1, 3 and 5) or an AP-site (lanes 2, 4 and 6) were purified and employed as substrates for *Bs*PolA-dependent DNA synthesis. As shown, this enzyme was able to polymerize DNA on the incision products of *Bs*EndoV on ds-DNA bearing Hx and X (A; lanes 2 and 5, respectively) but not on those containing U or an AP site (B; lanes 3 and 4, respectively). Positive controls were performed with *Pfu* Pol I (A, lanes 3 and 6; B, lanes 5 and 6). Negative controls were reactions lacking DNA polymerases (A, lanes 1 and 4; B, lanes 1 and 2). Shown beneath (**A)** and (**B**) are schematic representations of *B*sEndoV-incised and *Bs*PolA-extended products with Hx and X (2 and 3) and *Bs*EndoV-incised products of U and AP (2') lesions, respectively. Hx or X (◆); U or AP (■).

As noted above, *Bs*EndoV catalyzed the incision of ds-DNAsubstrates carrying deaminated bases or an AP site (Fig 2), and previous genetic evidences have shown that *Bs*PolA plays a critical role in the BER and NER pathways of *B*. *subtilis* [39]. Therefore, the mechanistic details of the *Bs*EndoV-dependent AER pathway were investigated further in *in vitro* repair assays that contained the post-incision repair products generated by *Bs*EndoV, after processing radioactive 19-mer ds-DNAs carrying Hx, X, U or an AP site. In these experiments the hydrolytic reactions of *Bs*EndoV on the four types of lesions were carried out for 2 h with saturating amounts of the enzyme allowing production of a 9-nt radioactive product with Hx and X and an 11-nt long product with U and AP (Fig 2A). The results with *Bs*PolA showed that this enzyme completed the incision repair event initiated by *Bs*EndoV on ds-DNA substrates containing Hx or X (Fig 4). In support of this contention, the 9-nt radioactive products that were free of Hx or X after the hydrolytic events catalysed by *Bs*EndoV, were efficiently extended by *Bs*PolA (Fig 4A, lanes 2 and 5) as well as by *Pfu* PolI, which was used as a positive control (Fig 4A, lanes 3 and 6). In marked contrast, *Bs*PolA was unable to extend the 11-nt radioactive products generated from the *Bs*EndoV-treated ds-DNA oligonucleotides containing uracil or AP-sites (Fig 4B, lanes 3, 4). Therefore, the single hydrolytic attack catalysed by *Bs*EndoV on DNA containing U or AP generates intermediates in which the 3'-OH group for *Bs*PolA-dependent extension are inaccessible.

### *Bs*EndoV operates in a common pathway with ExoA to counteract the mutagenic effects of uracil in DNA

**Fig 5:**
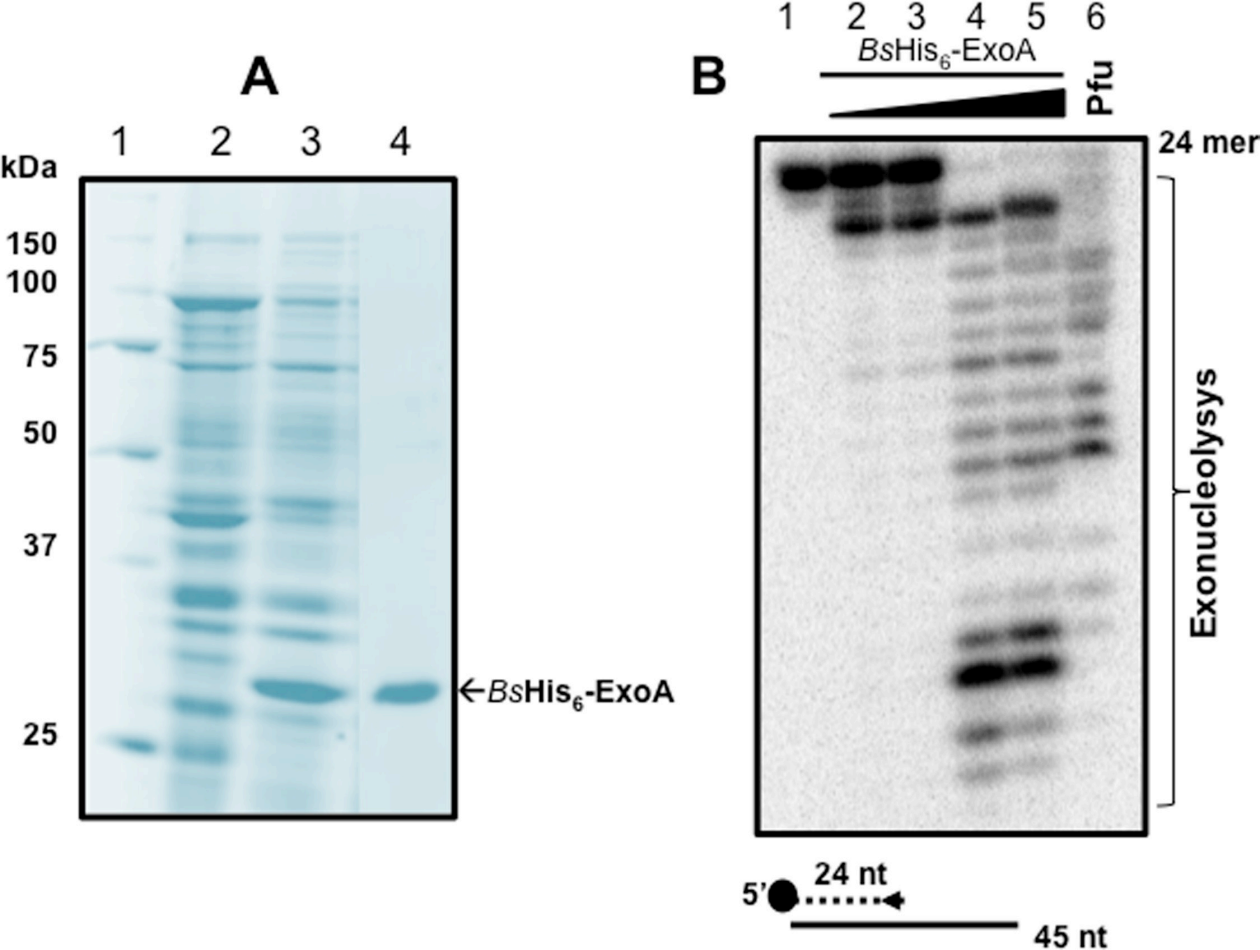
Purification and 3’➔5’ exonuclease activity of *Bs*ExoA. **(A)** SDS-PAGE analysis of His_6_-*Bs*ExoA induction and purification by IMAC as described in Methods. Lane 1, molecular weight markers; lane 2, cell lysate of non-induced *E*. *coli* PERM1311 strain; lane 3, IPTG-induced extract of *E*. *coli* PERM1311; and lane 4, protein eluted from the Ni-NTA column with 300 mM imidazole. **(B)** 3'➔5' exonuclease activity of *Bs*ExoA. Nuclease reactions were conducted with 0, 1, 10, 100 or 1000 nM of *Bs*ExoA (lanes 1–5) with a radiolabeled 24-mer-oligonucleotide hybridized with a non-radioactive 45-mer-oligonucleotide and incubated at 37°C as described in Methods. A positive control for DNA degradation was carried out with *Pfu* Pol I without dNTPs (lane 6). All reactions were separated by denaturing electrophoresis, exposed and analyzed as describe in Methods.

As noted above, *Bs*PolA failed to process the repair product generated from the attack of *Bs*EndoV on ds-DNA with U or an AP site. Therefore, we postulated that an additional incision event was required to remove these lesions before *Bs*PolA-dependent repair synthesis. The ability of *Pfu* PolI which possesses 3'➔5' exonuclease activity [40], to at least partially extend the U or AP-containing 11-nt radioactive products (Fig 4B, lanes 5 and 6), provided support for this hypothesis. In *B*. *subtilis*, ExoA, a homolog of *E*. *coli* Xth, has been previously purified and shown to possess AP-endonuclease and exonuclease activities, and plays an important role in the BER pathway [41, 42, 43]. To implement an *in vitro* repair assay to investigate whether ExoA is involved in the downstream repair events initiated by *Bs*EndoV on U and AP sites, we constructed an *E*. *coli* strain overproducing *B*. *subtilis* ExoA (*Bs*ExoA) carrying an N-terminal His_6_ tag (Fig 5A). The recombinant *Bs*ExoA had a molecular mass of ~30 kDa and was purified to homogeneity by metal affinity chromatography (Fig 5A). The purified *Bs*ExoA protein possessed exonuclease activity as demonstrated by its ability to hydrolyze a radiolabeled DNA in the 3'➔5' direction (Fig 5B, lanes 3 to 5). A similar result was obtained in a control reaction control that contained the *Pfu* Pol I under conditions that promote the 3'➔5' exonuclease activity of this enzyme (Fig 5B, lane 6).

### Repair intermediaries of uracil and AP sites generated by *Bs*EndoV are efficiently removed by *Bs*ExoA to allow *Bs*PolA-dependent DNA synthesis

**Fig 6:**
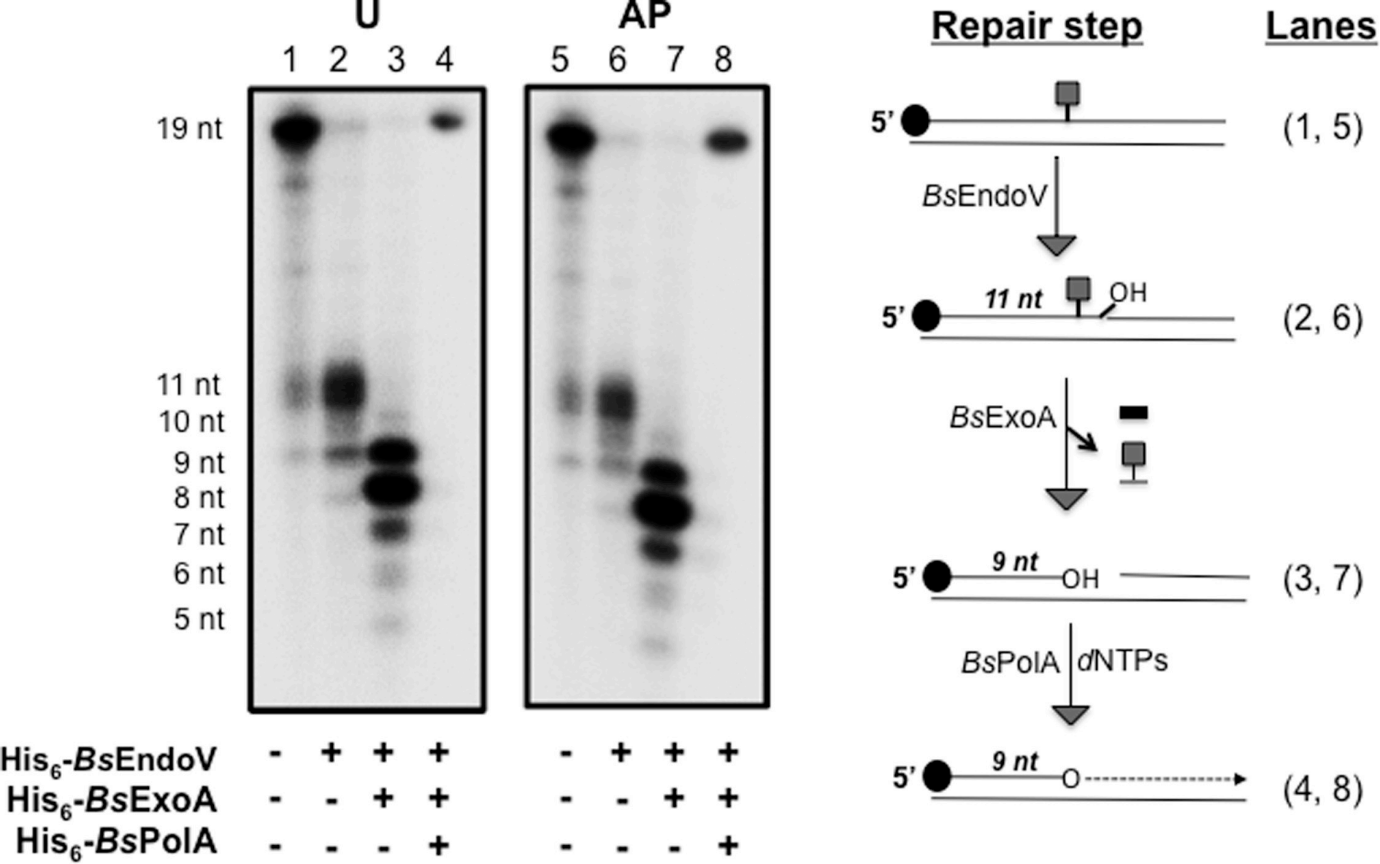
Capacity of *B*sExoA to remove *Bs*EndoV-repair intermediates and completion of repair by *Bs*PolA. 19-mer-ds-DNAs containing U (lane 1) or an AP-site (lanes 5) were subjected to incision by *Bs*EndoV to generate ds-DNA products with a single nick in the 11^th^ phosphodiester bond inaccessible to *Bs*PolA-dependent DNA synthesis (lanes 2 and 6). Addition of *Bs*ExoA to these repair products resulted in the efficient removal of nucleotides in the 3'➔5' direction, thus presumably removing the DNA damage (lanes 3 and 7). Finally, DNA synthesis was performed by *Bs*PolA completing the repair process (lanes 4 and 8). The distinct repair steps are schematically presented next to the autoradiograms with corresponding lane numbers. U or AP (■).

Having confirmed the activity of *Bs*ExoA, we evaluated its capacity to hydrolyze the blocking residues left after *Bs*EndoV processing of ds-DNA containing uracil or an AP-site. To this end, the radioactive-19bp DNAs containing uracil or an AP-site were subjected to incision by *Bs*EndoV as described above, generating 11 nt radioactive products (Fig 6, lanes 2 and 6 respectively). *Bs*ExoA was able to hydrolyze both latter radioactive products in the 3'➔5' direction, generating mostly 3-nt shorter radioactive oligonucleotides (Fig 6, lanes 3 and 7). After heat inactivation and phenol/chloroform extraction to eliminate *Bs*ExoA, the renatured residual repair products lacking the DNA lesions that presumably blocked the access of *Bs*PolA, were incubated with this replicase. As shown in Fig 6 (lanes 4 and 8), *Bs*PolA was able to successfully extend these ~ 8 nt repair intermediates, generating a product of 19-nt, thus completing the repair of these lesions. These results highlight the importance of *Bs*ExoA as an essential component of the *Bs*EndoV/*Bs*PolA-dependent excision repair pathway dealing with the deleterious effect of uracils and AP-sites in the *B*. *subtilis* genome.

### Contribution of EndoV, ExoA and PolA to spontaneous mutagenesis and damaging effects of base deamination in *B*. *subtilis*

**Fig 7:**
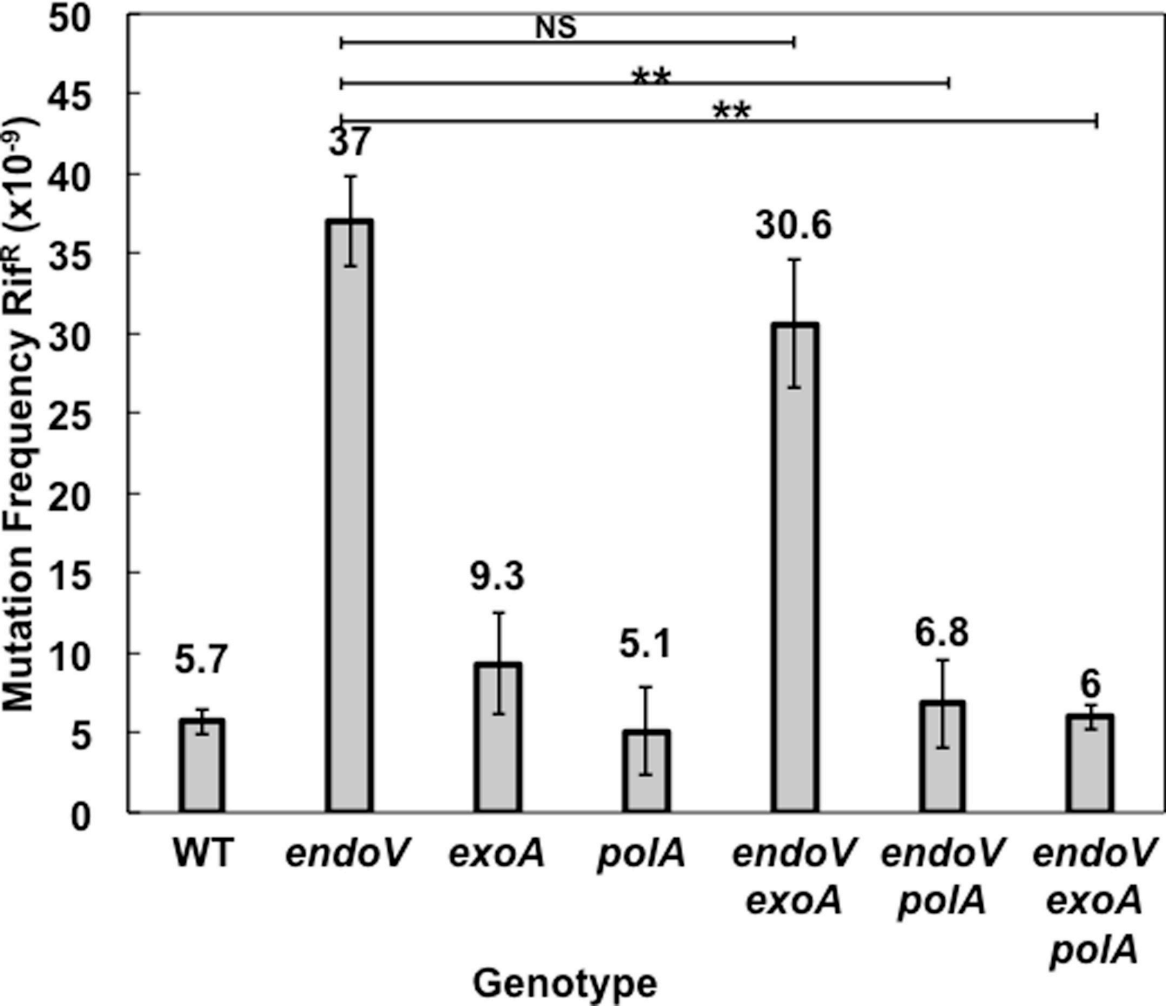
Frequencies of spontaneous mutation to Rif^r^ in cells of various *B*. *subtilis* strains. Cells of *B*. *subtilis* WT, *endoV*, *exoA*, *polA*, *endoV exoA*, *endoV polA* and *endoV exoA polA* strains were cultivated in liquid PAB medium, and spontaneous mutation frequencies to Rif^r^ were determined as described in Methods. Results represent the mean of three independent experiments. Error bars indicate the standard error of the mean (SEM). **, *P*< 0.01 (by the Mann-Whitney U test). NS, non significant.

All the *in vitro* data given above indicate that PolA, ExoA and EndoV can repair DNA with various base deaminations *in vitro*. However, an obvious question is whether these *in vitro* repair pathways also take place *in vivo*? To address this question, the spontaneous mutagenesis to rifampicin resistance (Rif^r^) was determined in *B*. *subtilis* cells deficient in *Bs*EndoV, PolA and ExoA proteins (Fig 7). Disruption of *exoA* did not significantly increase the spontaneous Rif^r^ mutation frequency of the *endoV* strain, suggesting that *Bs*EndoV and ExoA may work in a common pathway that counteracts spontaneous mutagenic events in *B*. *subtilis*. Notably, inactivation of *polA* significantly decreased the mutation frequency to Rif^r^ of *endoV* and *endoV exoA* strains suggesting that, in response to accumulation of DNA damage such as deaminated bases and AP sites, PolA can process these lesions in an error-prone manner.

**Fig 8:**
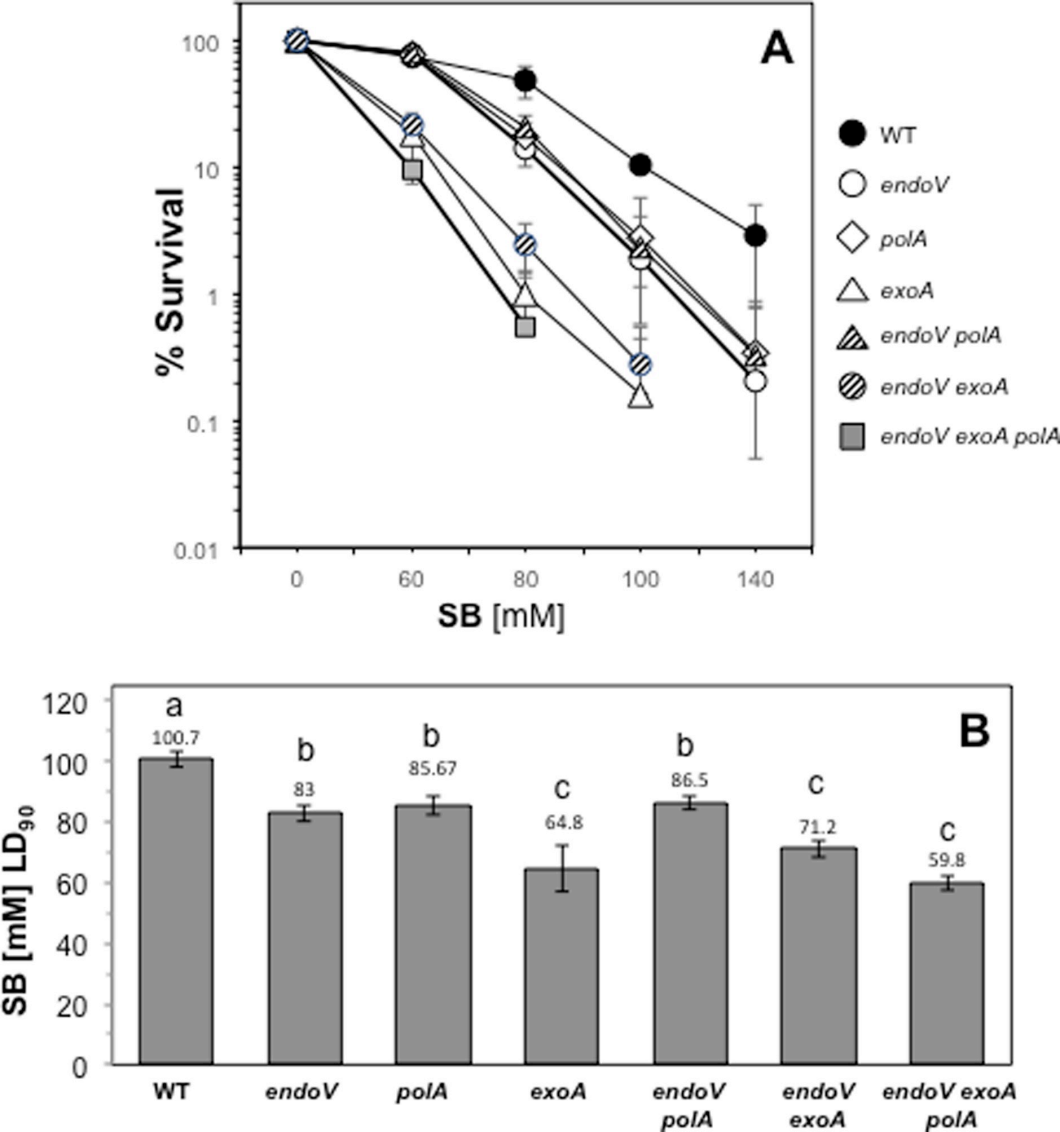
Effect of sodium bisulfite (SB) on survival of different *B*. *subtilis* strains. **(A).** Cells of *B*. *subtilis* WT, *endoV*, *exoA*, *polA*, *endoV polA*, *ywqL exoA* and *endoV exoA polA* strains were grown in liquid PAB medium to an OD_600nm_ of 0.5. Cells collected by centrifugation were washed with cold PBS and treated or not for 1 h at 25°C with distinct doses of SB. Cell survival after these treatments was determined by plating serial dilutions on solid LB medium, and colony-forming units were counted after 16 h of incubation at 37°C as indicated in Methods. (**B**). The LD_90_ was calculated for each strain from dose-response curves shown in **A**. Results are expressed as averages ± SD from at least three independent experiments. Error bars indicate the standard error of the mean (SEM); statistical significance (^a, b, c^) was determined by ANOVA followed by a Tukey test (*P* < .05).

We further investigated the contribution of *Bs*EndoV, *Bs*ExoA and *Bs*PolA to counteract the deleterious effects of base deamination in *B*. *subtilis*. To this end, strains with single and combined deficiencies in these proteins were challenged with increasing doses of the DNA deaminating agent sodium bisulfite (SB). As shown in Fig 8A and 8B, compared to the wild-type strain, whereas the *endoV* or *polA* mutants slightly increased SB susceptibility, the absence of ExoA greatly sensitized *B*. *subtilis* cells to this genotoxic agent. These analyses also revealed that disruption of *exoA* but not of *polA* significantly increased the SB susceptibility of the *endoV* strain (Fig 8A and 8B). Of note, the simultaneous inactivation of *exoA* and *polA* in the *endoV* strain did not significantly increase this strain’s SB susceptibility compared to the *endoV exoA* strain (Fig 8A and 8B). Taken together, these results strongly suggest that *Bs*EndoV, ExoA and *Bs*PolA all act to reduce the genotoxic and cytotoxic effects of base deamination in growing *B*. *subtilis* cells.

## Discussion

In this work, the substrate specificity as well the involvement of *Bs*PolA and *Bs*ExoA in the post incision events initiated by *Bs*EndoV on DNA containing deaminated and AP lesions was investigated. We further analyzed the *in vivo* roles of these enzymes in mutagenesis and resistance to a DNA-deaminating agent. Overall, our results revealed that *Bs*EndoV efficiently nicked DNA containing these lesions and together with *Bs*PolA completed the repair of Hx and X lesions, but an additional enzymatic excision event mediated by *Bs*ExoA was necessary to eliminate U and AP lesions through this alternative repair pathway.

To contend with the genotoxic and cytotoxic effects of base deamination, *B*. *subtilis* employs Ung and *Bs*EndoV [18–20]. Whereas a multiplicity of Ung proteins has been described in *E*. *coli* and mammals [44], *B*. *subtilis* has only one gene encoding an Ung [18]. However, as revealed a previous genetic study, *Bs*EndoV contributes more to avoid mutagenesis than Ung in this microorganism [18]. The biochemical properties of Ung, which specifically operates on uracil-containing DNA through the BER pathway, have been reported [34].

In the current work, the substrate specificity of *Bs*EndoV was evaluated employing radiolabelled DNA probes containing different types of DNA lesions. As described for its counterparts from *E*. *coli* and *T*. *maritima*, *Bs*EndoV exhibited enzyme activity against a wide range of damaged DNA substrates, including, Hx, X, U and AP sites. *Bs*EndoV had the same mode of action as that described for EndoV homologs from different species, hydrolyzing the second phosphodiester bond 3' to the four lesions tested (Fig 2A). However, while *Bs*EndoV shared substrate specificity and action properties with previously characterized EndoV proteins, some substantial differences were apparent. First, although *Bs*EndoV processed DNA substrates containing deaminated bases and AP sites *in vitro*, the cleavage preference showed a hierarchy of Hx = U>AP>X (Fig 2B and Table 1). These results differ from those with the *E*. *coli* and *Archeoglobus fulgidus* EndoV homologs, as the latter enzymes preferred Hx-containing DNA [21,45]. *Bs*EndoV also catalyzed an additional hydrolytic attack on the 5' phosphodiester bond flanking Hx and X lesions generating a 9-mer radioactive fragment free of damage; interestingly, neither the uracil- nor the AP site-containing DNAs suffered this secondary hydrolytic attack. A previous study revealed that the initial post-incision products generated by *T*. *maritima* EndoV can also undergo a second hydrolytic event when the enzyme is in excess. However, the second cleavage takes place on the complementary strand, generating a double-stranded break [46]. It has been reported that *E*. *coli* and *T*. *maritima* EndoV can use either Mg^2+^ or Mn^2+^ for efficient catalysis [11,46]. Interestingly, both the single and double nicking events catalyzed by *Bs*EndoV on U/AP and X/Hx containing DNA were performed when Mg^2+^ was present in the repair reactions (Fig 2A and 2C). However, the presence of Mn^2+^ in *Bs*EndoV reactions not only affected the specificity of the nicking event but also promoted unspecific hydrolytic cuts toward the 3' end of DNAs with the four lesions tested (Fig 2C). It has been shown that the presence of Mn^2+^ instead of Mg^2+^ uncovers a 3'➔5'-exonuclease activity in DNA-binding mutants of the EndoV homolog of *T*. *maritima* during repair of Hx-containing DNA [47]. However, under our experimental conditions, Mn^2+^ inhibited the *Bs*EndoV nicking at the 3' second phosphodiester bond near of all the damaged substrates and promoted a nonspecific incision (Fig 2C). Of note, a structural comparison between *Bs*EndoV and the crystal structures of the best characterized bacterial EndoVs revealed some important differences in key amino acids for recognition and processing of damage, including those in the stabilization wedge and damage recognition pocket involved in post-incision interactions with DNA, conserving only those residues necessary for the coordination of the Mg^2+^ ion necessary for the nucleophilic attack during phosphodiester bond cleavage (Fig 1A and 1B). We speculate that these structural changes in *Bs*EndoV could account for the different action of the enzyme when the DNA lesion is a Hx or X (two-nick mode), or a U or an AP site (one-nick mode). However, the molecular and mechanistic details of lesion recognition and incision remain to be elucidated.

In *B*. *subtilis*, PolA plays a central role in completing post incision/excision repair in the canonical NER, BER and MMR pathways [27,28,32]. Importantly, the results from an epistatic study, which analyzed the spontaneous Rif^r^ mutation frequency of *B*. *subtilis* cells deficient in *B*sEndoV, *Bs*PolA or both proteins, suggested that these proteins work in concert to repair DNA with deaminated bases and AP-sites (Fig 7). It must be pointed that under our experimental conditions *in vivo*, in the absence of *Bs*EndoV, *Bs*PolA is an error-prone processing enzyme, since the mutation rate decreased considerably when the *polA* gene was inactivated in the *endoV* strain (Fig 7). This mutagenic behavior of *Bs*PolA has been described previously [28], and it could be due to: i) the lack of proofreading by this enzyme due to the lack of a useful 3'➔5'exonuclease domain [23] and/or ii) the ability of *Bs*PolA to interact with error-prone DNA polymerases of the Y-family [27].

Based on these observations we initially considered *Bs*PolA to be a good candidate to complete repair event(s) initiated by *B*sEndoV on the four lesion-containing DNA substrates employed in this work. Indeed, the products resulting from the double nicking event catalyzed by *Bs*EndoV on DNA containing X and Hx were good substrates for *Bs*PolA. These results strongly support the concept that *Bs*EndoV and *Bs*PolA constitute a novel alternative pathway that eliminates X and Hx from the *B*. *subtilis* genome.

In contrast the nicked U- or AP-containing DNA oligomers generated by *Bs*EndoV action were not efficiently polymerized by *Bs*PolA (Fig 4). Therefore, we predicted that full repair following the nicking adjacent to U and an AP site by *Bs*EndoV would require a 3'➔5' exonuclease or a flap-endonuclease activity to eliminate a short DNA patch containing the lesion before DNA synthesis by *Bs*PolA. Notably, the lack of 3'➔5' exonuclease activity in *Bs*PolA [23,24] most probably prevented this enzyme from completing the repair of the U and AP intermediate products generated by *Bs*EndoV.

Interestingly, results from epistatic studies measuring Rif^r^ mutagenesis and susceptibility to a DNA-deaminating agent with *B*. *subtilis* mutants that combined deficiencies in *Bs*EndoV and the AP-endonuclease *Bs*ExoA suggest that both proteins did actually work in a common repair pathway in this bacterium (Figs 7 and 8). It has been shown that ExoA together with Nfo play important roles in DNA repair events in vegetative cells as well as in germinating/outgrowing spores of *B*. *subtilis* [41,42]. A previous report revealed that ExoA possesses a multiplicity of repair activities including hydrolysis of AP sites, ribonuclease H, 3'-phosphomonoesterase, as well as 3'➔5' exonuclease [40]. In agreement with this report, purified *Bs*ExoA efficiently hydrolyzed a radioactive ds-DNA substrate in the 3'➔5' direction (Fig 5).

**Fig 9:**
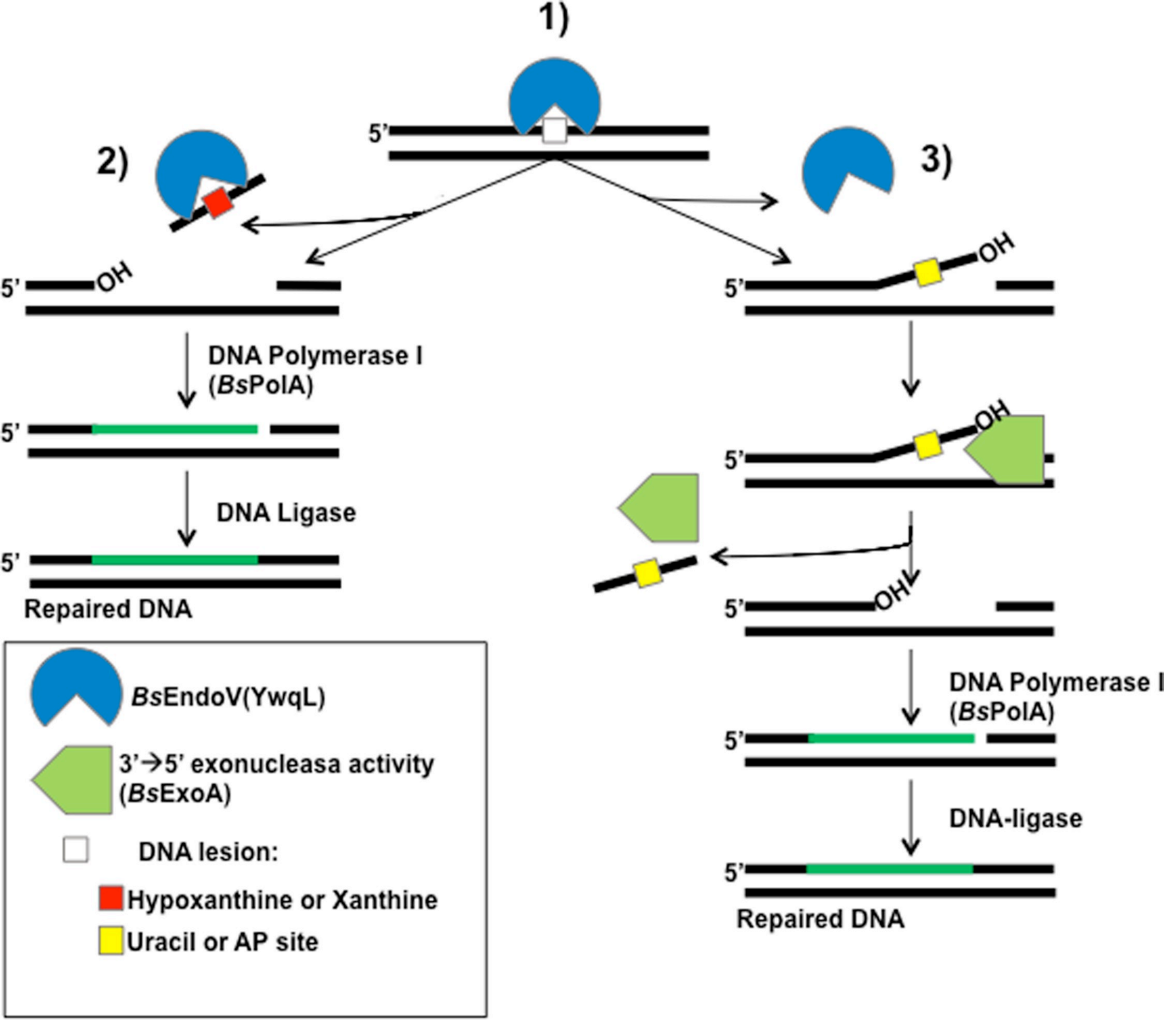
AER mechanism mediated by *Bs*EndoV, *Bs*ExoA and *Bs*PolA during removal of deaminated bases and AP sites in *B*. *subtilis* DNA. **(1)** *Bs*EndoV incises the second phospodiester bond downstream from a deaminated base or an AP site. Post-incision events are dependent on the type of damage. (2) *Bs*EndoV performs a second incision at the 5' side of the lesion when the deaminated bases are Hx or X, directly allowing DNA extension by *Bs*PolA. (3) Processing of U or an AP site initiated by *Bs*EndoV requires the subsequent activity of *Bs*ExoA before a *Bs*PolA-dependent DNA synthesis step. In both cases, a DNA ligase would be necessary to complete the AER events.

Since the products of the single incision event catalyzed by *Bs*EndoV on DNA carrying a single U or an AP lesion were not substrates for *Bs*PolA we hypothesized that the lesions made the 3'-OH of the hydrolyzed phosphodiester bond inaccessible for DNA synthesis. Indeed, the orientation of the single hydrolytic event catalyzed by *Bs*EndoV on these lesions (Figs 2 and 4) is such that DNA synthesis from these nicks will lead to futile repair events. As noted above, *Bs*PolA lacks 3'➔5' proofreading activity to eliminate the DNA stretch harboring a single U or AP lesion, but as demonstrated in this work, *Bs*ExoA proficiently catalyzed this excision event, and then *Bs*PolA efficiently extended the incision/excision repair intermediates resulting from the concomitant action of *Bs*EndoV and *Bs*ExoA thus completing the repair of these lesions *in vitro*. Overall, our results provide genetic and biochemical evidence for the existence of a novel AER pathway in *B*. *subtilis* that uses EndoV, ExoA and PolA to process a wide range of lesions, including, AP sites and deaminated bases (Fig 9).

## Materials and methods

### Bacterial strains, plasmids and growth conditions

Strains and plasmids used in this study are listed in S1 Table. Isolation of genomic DNA from *B*. *subtilis* 168 was as previously described [48], as were transformation and plasmid DNA isolation [49]. Liquid cultures of *E*. *coli* were grown in Luria-Bertani (LB) medium [50]. Liquid cultures of *B*. *subtilis* were grown in Difco antibiotic no. 3 (PAB) medium. When required, ampicillin (Amp) (100 μg mL^-1^), chloramphenicol (Cm) (25 μg mL^-1^), tetracycline (Tet) (15 μg mL^-1^), erythromycin (Er) (5 μg mL^-1^) or spectinomycin (Sp) (100 μg mL^-1^) was add to media and incubated at 37°C with vigorous aeration; growth was followed by measuring cultures’ optical density at 600 nm (OD_600nm_).

### Sequence and structure analysis of EndoV proteins

Alignments of amino acid sequences from *B*. *subtilis* EndoV, *E*. *coli* Nfi and *T*. *maritima* Nfi were performed with the sequence analyser Geneious software. Crystal structures of *B*. *subtilis* (YwqL) [51], *E*. *coli* (Nfi) [37] and *T*. *maritima* (Nfi) [52] EndoVs with the accession numbers in protein data bank 3GA2, 4XPU and 3HD0, respectively, were visualized and coloured for structural and amino acid comparison.

### Design of DNA constructs for protein expression

To overexpress *Bs*EndoV, *Bs*ExoA and *Bs*PolA, the ORFs of *endoV*, *exoA* and *polA* lacking the start and stop codons were amplified by PCR using specific oligonucleotide primers carrying the appropriate restriction enzyme sequences (S2 Table) and the PCR products ligated between the multiple cloning sites (MCS) of the expression vector pQE30 (QIAGEN Inc., Valencia, CA). The resulting recombinant constructs carrying respective in-frame hexahistidine tags on the 5´-ends of *polA*, *endoV* and *exoA* (S1 Table) were obtained in *E*. *coli* XL10-Gold (Tc^R^). All PCR reactions were performed with high fidelity Vent DNA polymerase (New England Bio Labs, Ipswich, MA) and chromosomal DNA from *B*. *subtilis* 168.

### Purification of recombinant proteins

Strains containing each pQE30 construct were grown at 37°C in LB medium supplemented with ampicillin and tetracycline. At an OD_600nm_ of 0.5, gene expression was induced with 0.25 mM isopropyl-ß-D-thiogalactopyranoside (IPTG) and growth continued for 4 h at 28°C for *endoV*, 3h at 30°C for *exoA* and 2h at 37°C for *polA*. Cells were collected by centrifugation (4800 × *g*, 10 min), washed twice with 10 mL of lysis buffer (see below) and finally resuspended in 20 mL of this buffer. The cell suspensions were treated with lysozyme (0.2 mg/mL), incubated for 30 min at 37°C, disrupted by sonication, and the extracts were centrifuged (15 000 × *g*, 20 min, 4°C) to remove cells debris. Cell-free crude extract was applied to a 5 mL Ni-nitrilotriacetic acid (NTA) agarose column (QIAGEN Inc., Valencia, CA), previously equilibrated with lysis buffer. The column was washed with 30 volumes of wash buffer (see below) before eluting the protein bound to the resin with 5 mL of elution buffer (see below). Eluted fractions were dialyzed overnight in storage buffer (see below), aliquoted and stored at -20°C until use. The composition of buffers used to purify *Bs*EndoV were: lysis buffer (50 mM Tris-HCl [pH 7.5], 300 mM NaCl, 10 mM imidazole, 0.03% Tween 20); wash buffer (50 mM Tris-HCl [pH 7.5], 300 mM NaCl, 20 mM imidazole, 0.03% Tween 20); elution buffer (50 mM Tris-HCl [pH 7.5], 300 mM NaCl, 250 mM imidazole, 0.03% Tween 20, 5mM DTT); storage buffer (50 mM Tris-HCl [pH 7.5], 100 mM NaCl, 5mM DTT, 50% glycerol). The composition of buffers to purify *Bs*ExoA and *Bs*PolA were: lysis buffer (50 mM NaH_2_PO_4_ [pH 8], 300 mM NaCl, 10 mM imidazole, 0.05% Tween 20); wash buffer (50 mM NaH_2_PO_4_ [pH 8], 300 mM NaCl, 50 mM imidazole, 0.05% Tween 20); elution buffer (50 mM NaH_2_PO_4_ [pH 8], 300 mM NaCl, 300 mM imidazole, 0.05% Tween 20); dialysis/storage buffer (50 mM NaH_2_PO_4_ [pH 8], 100 mM NaCl, 5mM DTT, 50% glycerol). Aliquots of the cell homogenate and the column flow-through as well as the eluted bound fractions were analysed by sodium dodecyl sulphate-polyacrylamide gel electrophoresis (SDS-PAGE) and gels were stained as previously described [53].

### Substrates and enzymatic assays to determine endonuclease activity of recombinant *Bs*EndoV

The enzymatic acivity of recombinant *Bs*EndoV was assessed against a radioactive double-stranded 19-mer nucleotide. For this purpose, 100 nM of the oligonucleotide 5'-GCACCGGAC**X**GAGGCGACG (Sigma-Aldrich, The Woodlands, TX) containing a single U, Hx, X or AP-site at nt 10 denoted by underlined “**X**” above was 5' end-labelled with [γ-^32^P] ATP and T4 polynucleotide kinase (New England Bio-Labs, Ipsiwch, MA). The radioactive oligonucleotide was annealed to the complementary oligonucleotide 5'-CGTCGCCTC**Y**GTCCGGTGC (where the underlined “Y” was substituted with A as a complementary base for U or an AP-site, T as a complementary base for Hx and by C as a complementary base for X. The three complementary oligonucleotide primers were used at a final concentration of 10 nM in annealing buffer (5 mM Tris-HCl [pH 7.5], 1 mM NaCl). For proper annealing of complementary strands, the samples were heated for 5 min at 95°C and cooled slowly to room temperature [54]. 10 nM of radioactive ds-DNA containing the lesion were used as substrates in 50 μL reactions that included, 5 mM Tris-HCl [pH 7.5], 100 nM of purified *Bs*EndoV, 10 μg/mL BSA and 5 mM DTT. The divalent metal ion cofactor, either MgCl_2_ or MnCl_2_, was at 5 mM. All reactions were incubated at 37°C and samples were collected at different times and diluted 1:1 (v/v) with stop buffer (95% formamide, 20 mM EDTA, 0.1% xylene cyanol and 0.01% bromophenol blue). Aliquots of 5 μL were run on electrophoresis through 8 M urea-17% polyacrylamide gels and analyzed by phosphorimagery on a personal molecular imager (Bio-Rad Laboratories, Hercules, CA). Densitometry analysis of reaction products was performed using the software ImageJ 1.47n (http://imagej.nih.gov/ij/).

### Determination of spontaneous mutagenesis and treatment of cells with sodium bisulfite (SB)

Mutation rates to Rif^r^ were determined as follows. Cultures of each strain grown overnight were inoculated into flasks containing fresh PAB medium and OD_600nm_ monitored until reaching 0.5 and then further incubated at 37°C for 12 h with vigorous aeration. Mutation frequencies were calculated by spreading aliquots on six LB plates supplemented with rifampin (Rif) (10 μg mL^-1^) as well as spreading aliquots of serial dilutions on LB plates to determine total viable counts. Rif^r^ colonies were counted after 48 h of incubation at 37°C. Differences in mutagenesis rates between *B*. *subtilis* strains were calculated with the non-parametric Mann-Whitney *U*-test. *P*<0.01 denoted significant differences. Analyses were done using Minitab 17 software.

Cultures of the different strains were propagated in liquid PAB medium to an OD_600nm_ of 0.5. Cells of each culture collected by centrifugation were washed with cold phosphate-buffered saline (PBS; 0.7% Na_2_HPO_4_, 0.3% KH_2_PO_4_, 0.4% NaCl [pH 7.5]). Cell aliquots in PBS were treated or not with 60, 80, 100 or 140 mM (final concentration) of SB for 1 h at 25°C. Cell survival after these treatments was determined by plating serial dilutions on solid LB medium, and colony-forming units were counted after 24 h of incubation at 37°C. Statistical significance between strains was determined by performing one-way ANOVA followed by Tukey’s post-hoc analysis. Significance was set at *P* < 0.05.

### DNA polymerase activity of *Bs*PolA

To determine the DNA synthesis capacity of purified *Bs*PolA *in vitro*, a 45-mer DNA oligonucleotide (5'-CCTTGGCACTAGCGCAGGGCCAGTTAGGTGGGCAGGTGGGCTGCG) was hybridized as described above to the complementary 5’-end [γ-^32^P] labelled 24-mer oligonucleotide 5'-CGCAGCCCACCTGCCCACCTAACT-3'. Reactions containing 20 nM of ds-DNA and purified *Bs*PolA (0, 1, 10, 100 or 1000 nM) were incubated at 37°C in polymerization buffer (20 mM Tris-HCl [pH 7.5], 5 mM MgCl_2_, 5 mM DTT) supplemented with 250 μM of each dNTP. All reactions were incubated at 37°C for 10 min and then diluted 1:1 (v/v) with stop buffer. A positive control for DNA polymerization used *Pfu* DNA polymerase following the instructions of the supplier (Monserate Biotechnology Group, San Diego, CA). Reactions products were resolved by 17% denaturing polyacrylamide gels and analysed by phosphorimagery.

### Exonucleolytic activity of *Bs*ExoA

The ability of *Bs*ExoA to degrade DNA in the 3'➔5' direction was evaluated at 37°C in 50 μL reactions containing 0, 1, 10, 100 and 1000 nM *Bs*ExoA, 20 nM of a 24/45-nt annealed radioactive ds-DNA probe described above, 5 mM Tris-HCl [pH 7.5], 5 mM MgCl_2_ and 100 mM NaCl. The reaction was diluted 1:1 (v/v) with stop buffer at the end of the incubation. A positive control for exonuclease in the 3'➔5' direction used a unit of *Pfu* Pol I in a reaction lacking dNTP’s. All reaction products were resolved by 17% denaturing polyacrylamide gels and analyzed by phosphorimagery.

### *In vitro* reconstitution of an alternative excision repair pathway to process deaminated bases and AP sites

To assess the participation of ExoA and PolA as part of the DNA repair pathway initiated by *Bs*EndoV, 100 nM of each ds-DNA containing the various lesions was treated in 50 μL reactions with a saturating amount of *Bs*EndoV (1μM) that included 5 mM Tris-HCl [pH 7.5], 1mM DTT, 10 μg/mL BSA and 5 mM MgCl_2_. The reactions were incubated at 37°C for 1h. After confirming the full cleavage of the DNA probes by the recombinant protein, the intermediate products were extracted from the reaction mixtures with phenol:chloroform (25:25 v/v), precipitated with 10 volumes of absolute ethanol and 100 nM sodium acetate and dissolved in nuclease-free water. 40 μL reactions containing 20 nM of each [^32^P]-labelled protein-free ds-DNA products and 100 nM *Bs*PolA in polymerization buffer were incubated for 30 min at 37°C. When required, prior to the DNA synthesis, 20 nM of the *Bs*EndoV-incised ds-DNA containing U or AP-sites were treated with 10 nM of *Bs*ExoA for 5 min in the same polymerization buffer; *Bs*ExoA was inactivated by heat at 70°C during 10 min before DNA synthesis. Where indicated, the exonuclease activity of *Pfu* Pol I, incubated under the manufacturers recommendations (Monserate Biotechnology Group), was used as a control. Reaction products were resolved by 17% denaturing polyacrylamide gels and analysed by phosphorimagery.

## Supporting information

S1 Table Strains and plasmids used in this study. (PDF)

S2 Table Oligonucleotides used to amplify and clone the ORFs of *endoV*, *exoA* and *polA* into pQE30. (PDF)

S1 Fig Incapability of *Bs*EndoV to operate over a 19-mer ds-DNA free of lesions. (DOCX)
