# Supplementary material for: YwqL (EndoV), ExoA and PolA act in a novel alternative excision pathway to repair deaminated DNA bases in Bacillus subtilis
Source: PLoS One. 2019 Feb 6;14(2):e0211653. doi: 10.1371/journal.pone.0211653 (PMC6364969; doi:10.1371/journal.pone.0211653)
Supplement: S1 Table — (PDF) [file pone.0211653.s001.pdf]

YwqL (EndoV), ExoA and PolA act in a novel alternative excision pathway to repair a wide spectrum of DNA lesions in *Bacillus subtilis*

Adriana G. Patlán[1&], Víctor M. Ayala-García[1&#], Luz I. Valenzuela-García[1], Jimena Meneses-Plascencia[1], Pedro L. Vargas-Arias[1], Marcelo Barraza-Salas[2], Peter Selow[3], Luis G. Brieba[4] and Mario Pedraza-Reyes[1]*

[1]Departamento de Biología, Universidad de Guanajuato, Noria Alta, Guanajuato, Guanajuato, México

[2]Facultad de Ciencias Químicas, Universidad Juárez del Estado de Durango, Durango, Durango, México.

[3]Department of Molecular Biology and Biophysics, UConn Health, Farmington, Connecticut, USA

[4]Langebio-Cinvestav Sede Irapuato, Km. 9.6 Libramiento Norte. Carretera Irapuato-León, Irapuato, Guanajuato, México.

Short title: YwqL(EndoV)-dependent repair of deaminated bases and AP-sites in *B. subtilis*

[#] Current Address: Facultad de Ciencias Químicas, Universidad Juárez del Estado de Durango, Durango, Durango, México.

* Corresponding author

E-mail: pedrama@ugto.mx (MPR)

[&]The first two authors contributed equally to this work

Table S1. Strains and plasmids used in this study.

Strain or Plasmid	Genotype or description[a]	Source[b]
B. subtilis		
168	*trpC*$^{-}$ (wild-type)	Laboratory stock
AMP100	YB955 *polA::sp*; Sp^R	(28)
PERM383	*exoA::tc* Tc^R	pPERM374→168
PERM791	*endoV::lacZ*; Er^R	(18)
PERM1213	*endoV::lacZ; exoA::tc*; Er^R Tc^R	PERM791→PERM383
PERM1521	*polA::sp*; Sp^R	AMP100→168
PERM1554	*endoV::lacZ*; *polA::sp*; Er^R Sp^R	PERM1521→PERM791
PERM1681	*endoV::lacZ; exoA::tc; polA::sp* Er^R Tc^R Sp^R	AMP100→PERM1213
E. coli		
XL10-Gold Tc^R	Tc^R Δ(*mcrA*)183 Δ(*mcr*CB-*hsd*SMR-mrr)173 *end*A1 *sup*E44 *thi*-1 *recA1 gyrA96 relA1 lac* Hte [F′ *pro*AB *lac*IqZΔM15 Tn10 (Tc^R) Amy Cm^R].	Stratagene
PERM1071	XL10-Gold Tc^R containing plasmid pQE30 with the ORF of *endoV* (Amp^R)	This study
PERM1311	XL10-Gold Tc^R containing plasmid pQE30 with the ORF of *exoA* (Amp^R)	This study
PERM1367	XL10-Gold Tc^R containing plasmid pQE30 with the ORF of *polA* (Amp^R)	This study
Plasmids		
pQE30	Expression vector containing T_5 promoter that enables expression of 6xHis-tagged proteins (Amp^R)	QIAGEN
pPERM374	pDG1515 with 537-bp EcoRI-HindIII PCR product containing the 3' region of *exoA*; Amp^R Tc^R	(41)
pPERM1071	pQE30 containing the ORF (711 bp) of *endoV* (Amp^R)	This study
pPERM1311	pQE30 containing the ORF (750 bp) of *exoA* (Amp^R)	This study
pPERM1367	pQE30 containing the ORF (2637 bp) of *polA* (Amp^R)	This study

[a] Selection markers: Amp, ampicillin; Cm, chloramphenicol; Er, erythromycin; Sp, spectinomycin; Tc, tetracycline.

[b] X→Y indicates that strain Y was transformed with DNA from source X.
