# Supplementary material for: YwqL (EndoV), ExoA and PolA act in a novel alternative excision pathway to repair deaminated DNA bases in Bacillus subtilis
Source: PLoS One. 2019 Feb 6;14(2):e0211653. doi: 10.1371/journal.pone.0211653 (PMC6364969; doi:10.1371/journal.pone.0211653)
Supplement: S2 Table — (PDF) [file pone.0211653.s002.pdf]

YwqL (EndoV), ExoA and PolA act in a novel alternative excision pathway to repair a wide spectrum of DNA lesions in *Bacillus subtilis*

Adriana G. Patlán[1&], Víctor M. Ayala-García[1&#], Luz I. Valenzuela-García[1], Jimena Meneses-Plascencia[1], Pedro L. Vargas-Arias[1], Marcelo Barraza-Salas[2], Peter Selow[3], Luis G. Brieba[4] and Mario Pedraza-Reyes[1]*

[1]Departamento de Biología, Universidad de Guanajuato, Noria Alta, Guanajuato, Guanajuato, México

[2]Facultad de Ciencias Químicas, Universidad Juárez del Estado de Durango, Durango, Durango, México.

[3]Department of Molecular Biology and Biophysics, UConn Health, Farmington, Connecticut, USA

[4]Langebio-Cinvestav Sede Irapuato, Km. 9.6 Libramiento Norte. Carretera Irapuato-León, Irapuato, Guanajuato, México.

Short title: YwqL(EndoV)-dependent repair of deaminated bases and AP-sites in *B. subtilis*

[#] Current Address: Facultad de Ciencias Químicas, Universidad Juárez del Estado de Durango, Durango, Durango, México.

* Corresponding author

E-mail: pedrama@ugto.mx (MPR)

[&]The first two authors contributed equally to this work

Table S2. Oligonucleotides employed to amplify and clone the *B. subtilis* ORFs of *endoV*, *exoA* and *polA* into pQE30.

Amplified gene	Oligonucleotide sequence*	Restriction site
endoV	5'-GCGGATCCAAGGTATTTGATGTGCAT (forward)	*Bam*HI
	5'-CCAAGCTTCACGTGATTTTTCTGATA (reverse)	*Hin*dIII.
exoA	5'-GCGGATCCAAGTTGATTTCATGG (forward)	*Bam*HI
	5'-GCAAGCTTTATATTGATGATAAG (reverse)	*Hin*dIII.
polA	5'-GCGGATCCACGGAACGAAAAAAATTA (forward)	*Bam*HI
	5'-GCGGTACCTTTCGCATCGTACCAAGA (reverse)	*Kpn*I

*underlined letters indicate restriction site.
