# Supplementary material for: YwqL (EndoV), ExoA and PolA act in a novel alternative excision pathway to repair deaminated DNA bases in Bacillus subtilis
Source: PLoS One. 2019 Feb 6;14(2):e0211653. doi: 10.1371/journal.pone.0211653 (PMC6364969; doi:10.1371/journal.pone.0211653)
Supplement: S1 Fig — (DOCX) [file pone.0211653.s003.docx]

**SUPPLEMENTARY INFORMATION**

**YwqL (EndoV), ExoA and PolA act in a novel alternative excision pathway to repair deaminated DNA bases in *Bacillus subtilis***

Adriana G. Patlán^1&^, Víctor M. Ayala-García^1&#^, Luz I. Valenzuela-García^1^, Jimena Meneses-Plascencia^1^, Pedro L. Vargas-Arias^1^, Marcelo Barraza-Salas^2^, Peter Selow^3^, Luis G. Brieba^4^ and Mario Pedraza-Reyes^1^*

^1^Departamento de Biología, Universidad de Guanajuato, Noria Alta, Guanajuato, Guanajuato, México

^2^Facultad de Ciencias Químicas, Universidad Juárez del Estado de Durango, Durango, Durango, México.

^3^Department of Molecular Biology and Biophysics, UConn Health, Farmington, Connecticut, USA

^4^Langebio-Cinvestav Sede Irapuato, Km. 9.6 Libramiento Norte. Carretera Irapuato-León, Irapuato, Guanajuato, México.

**Short title:** YwqL(EndoV)-dependent repair of deaminated bases and AP-sites in *B. subtilis*

^#^ Current Address: Facultad de Ciencias Químicas, Universidad Juárez del Estado de Durango, Durango, Durango, México.

* Corresponding author

E-mail: pedrama@ugto.mx (MPR)

^&^The first two authors contributed equally to this work

**SUPPLEMENTAL S1 Legend**

**S1 Fig. Incapability of *Bs*EndoV to operate over a 19-mer ds-DNA free of lesions.** A [^32^P]-labeled 19-mer-DNA (at 10 nM concentration) free of lesions was annealed to its complementary non-radioactive oligonucleotide and incubated separately with 100 nM of *Bs*EndoV in presence of MgCl_2_ as described in Methods, and samples were collected at indicated times. An additional reaction (column 1) contained the labeled oligonucleotide with U incubated with commercial uracil-DNA glycosylase (Ung) and AP-endonuclease (Nfo) in order to estimate the size of the undamaged DNA substrate. The reactions were separated by denaturing electrophoresis, exposed and analyzed by phosphorimagery.

**SUPPLEMENTAL S1 Figure**
